# Sphingomyelinase D Activity in Model Membranes: Structural Effects of *in situ* Generation of Ceramide-1-Phosphate

**DOI:** 10.1371/journal.pone.0036003

**Published:** 2012-04-25

**Authors:** Roberto P. Stock, Jonathan Brewer, Kerstin Wagner, Blanca Ramos-Cerrillo, Lars Duelund, Kit Drescher Jernshøj, Lars Folke Olsen, Luis A. Bagatolli

**Affiliations:** 1 Instituto de Biotecnología, Universidad Nacional Autónoma de México, Cuernavaca, Morelos, Mexico; 2 Membrane Biophysics and Biophotonics Group/MEMPHYS, Department of Biochemistry and Molecular Biology, Center for Biomembrane Physics, University of Southern Denmark, Odense, Denmark; 3 MEMPHYS, Department of Physics, Chemistry and Pharmacy, Center for Biomembrane Physics, University of Southern Denmark, Odense, Denmark; 4 Cellular Complexity Group (CelCom), Department of Biochemistry and Molecular Biology, University of Southern Denmark, Odense, Denmark; Consejo Superior de Investigaciones Cientificas, Spain

## Abstract

The toxicity of *Loxosceles* spider venom has been attributed to a rare enzyme, sphingomyelinase D, which transforms sphingomyelin to ceramide-1-phosphate. The bases of its inflammatory and dermonecrotic activity, however, remain unclear. In this work the effects of ceramide-1-phosphate on model membranes were studied both by *in situ* generation of this lipid using a recombinant sphingomyelinase D from the spider *Loxosceles laeta* and by pre-mixing it with sphingomyelin and cholesterol. The systems of choice were large unilamellar vesicles for bulk studies (enzyme kinetics, fluorescence spectroscopy and dynamic light scattering) and giant unilamellar vesicles for fluorescence microscopy examination using a variety of fluorescent probes. The influence of membrane lateral structure on the kinetics of enzyme activity and the consequences of enzyme activity on the structure of target membranes containing sphingomyelin were examined. The findings indicate that: 1) ceramide-1-phosphate (particularly lauroyl ceramide-1-phosphate) can be incorporated into sphingomyelin bilayers in a concentration-dependent manner and generates coexistence of liquid disordered/solid ordered domains, 2) the activity of sphingomyelinase D is clearly influenced by the supramolecular organization of its substrate in membranes and, 3) *in situ* ceramide-1-phosphate generation by enzymatic activity profoundly alters the lateral structure and morphology of the target membranes.

## Introduction

Spiders of the genus *Loxosceles* are considered of medical importance because they are capable of causing severe local and occasionally systemic envenomation in humans [1]–[3]. The venom of *Loxosceles* spiders contains an unusual enzyme responsible for the dermonecrotic local lesions characteristic of the envenomation syndrome (cutaneous loxoscelism) and its rarer but more severe systemic effects [4]–[6]. An extensive biochemical characterization of this enzyme is that of Lee and Lynch [7], who established that it is a phospholipase which can hydrolyze sphingomyelin (SM) as well as an array of lysoglycerophospholipids with various polar head groups, but not double chain glycerophospholipids or sphingosylphosphorylcholine. The enzyme has been often referred to as sphingomyelinase D (SMase D, SMD) or, as Lee and Lynch suggested, phospholipase D by virtue of its wider spectrum of lipid substrates. Since this study deals with the effects of the action of this enzyme on model sphingomyelin- containing membrane systems, we will refer to it as SMD. When acting on SM, the enzyme releases choline and ceramide-1-phosphate (Cer-1-P). Although it is reasonable to suppose that other venom components can influence the outcome of envenomation, it has been convincingly demonstrated that SMD is both necessary and sufficient to elicit the characteristic dermonecrotic lesions [8], which typically follow a massive inflammatory response that has by now a well described natural history [9]. Morphological changes [10] and increased susceptibility to complement-mediated hemolysis of red blood cells resulting from SMD action have been reported [11], demonstrating that SM is available for enzymatic action. van Meeteren et al. have argued that the lysophospholipase activity of the enzyme on circulating lysophospholipids underlies the inflammatory and dermonecrotic effects of the venom [12] and find a role for the sphingomyelinase D activity unlikely [13]. Dragulev et al., however, have shown that recombinant SMD by itself can elicit production of proinflammatory factors in the absence of any source of exogenous lysophospholipids [14]. The latter favor the idea that the sphingomyelinase D activity of the enzyme is important in its overall toxicity with the implication that ceramide-1-phosphate plays a key role in the cellular response that leads to the clinical manifestations. These proposals are not mutually exclusive [15] and SMD has been recently suggested as a useful new probe in the study of the effects of SM metabolism at the cellular level [16].

Over the last decade an increasing amount of study has been devoted to the physicochemical characterization of sphingolipids, their metabolism and involvement in an important number of cellular processes [17]–[19]. Although sphingomyelins are very abundant in many cellular membranes, the level of the simple phosphosphingolipid Cer-1-P is very low [20]. In mammalian cells Cer-1-P is synthesized by ceramide kinase from ceramide (Cer) and hydrolyzed to ceramide by a ceramide-1-phosphatase activity. Its low abundance, coupled to by now well established synthetic and degradative pathways, have led to the proposal that Cer-1-P levels are tightly regulated and that this simplest of phosphosphingolipids is an important signaling molecule [21]; fluctuations in Cer-1-P levels have been associated to a number of cellular processes such as phagocytosis [18], ion channel activity [22], inflammation [23], cell survival and tumorigenesis [24], [25]. Although the exact role of sphingolipids in these phenomena remains to be established two general views, not necessarily exclusive, are espoused: in one, sphingolipids are viewed as second messengers responsible for triggering the action of effectors but without a direct role as lipid species within distinct lipid supramolecular assemblies; in another view, their accumulation confers particular supramolecular properties to the membranes in which they are incorporated (permeability, lateral organization in terms of packing and/or curvature) in ways that elicit cellular responses by local environmental changes [26]. Distinguishing between these possibilities is not a trivial task, as a description of the effects of supramolecular lipid structure on cellular activities requires a general understanding of how lipid supramolecular/interfacial properties impinge on a wide array of cellular processes. In the particular case of Cer-1-P, a more specific understanding of the properties of membranes containing this lipid is required. Basic aspects of the membrane organization and ionization behavior of palmitoyl Cer-1-P, both pure and mixed with selected glycerophospholipids, have been described [27]. This article shows that the main transition temperature of Cer-1-P is considerably higher than that of its parent SM but, unlike ceramide or the structurally similar lysophosphatidic acid, pure Cer-1-P is capable of forming lamellar phases. Somewhat paradoxically, Cer-1-P can induce negative curvature at low concentrations and destabilize lamellar phases of particular glycerophospholipids [27].

The general goal of this study was to establish whether SMD activity could cause gross changes in the supramolecular properties of SM-containing membranes. To this end, the study was divided into three parts. The first focused on the effects of Cer-1-P on the supramolecular organization of SM-based model membranes under equilibrium conditions (i.e. in the absence of enzyme). Second, the effects of substrate supramolecular structure and composition on SMD activity were addressed. Finally, some structural/morphological effects resulting from the accumulation of Cer-1-P by SMD action in model membranes were characterized. The kinetics of SMD activity were studied using SM substrates with different states of organization, such as micelles and unilamellar vesicles. Structural changes in SM-containing large and giant unilamellar vesicles upon enzymatic action were also studied by a variety of optical methods.

## Materials and Methods

### Lipids and reagents

N-lauroyl-D-*erythro*-sphingosylphosphorylcholine (C_12_SM), egg sphingomyelin (egg SM, 86% N-hexadecanoyl-D-*erythro*-sphingosylphosphorylcholine), N-lauroyl-ceramide-1-phosphate (C_12_Cer-1-P), N-palmitoyl-ceramide-1-phosphate (C_16_Cer-1-P), 1,2-dioleoyl-*sn*-glycero-3-phosphocholine (DOPC), 1-palmitoyl-2-oleoyl-*sn*-glycero-3-phosphocholine (POPC), 1-lauroyl-2-hydroxy-*sn*-glycero-3-phosphocholine (C_12_LPC) and cholesterol, where purchased from Avanti Polar Lipids (Alabaster, AL, USA). Unless specified otherwise, all lipids were in dry powder form and were used without further purification. After dissolving to either 10 mM in chloroform (C_12_ and egg SM) or chloroform∶methanol∶water (80∶20∶2 v/v/v, C_12_ and C_16_Cer-1-P) lipid concentration was determined by phosphorus analysis [28], [29]. The fluorescent probes 1,1′-dioctadecyl-3,3,3′,3′-tetramethylindocarbocyanine perchlorate (DiIC_18_) and 6-dodecanoyl-2-dimethylaminonaphthalene (LAURDAN) were from Invitrogen (Molecular Probes, Copenhagen, Denmark). Atto647-conjugated 1,2-dipalmitoyl-sn-glycero-3-phosphoethanolamine (Atto647-DPPE) was from AttoTec, Siegen, Germany. All general reagents (Triton X-100, solvents, buffers and salts) were from Sigma-Aldrich (Copenhagen, Denmark).

### Differential scanning calorimetry (DSC)

Suspensions of 2 or 5 mM C_12_SM, C_12_Cer-1-P, egg SM, C_16_Cer-1-P were prepared by hydration of dry lipid films in phosphate buffered saline (PBS), 2 mM MgCl_2_, pH 7.4, by vortexing for 90 minutes at 75°C. Differential scanning calorimetry (DSC) for the C_12_SM was performed using a Calorimetric Sciences Corp. model 4100 MC-DSC (TA Instruments, Lindon, UT, USA). The samples were allowed to equilibrate and then scanned from −10 to 85°C with a scan speed of 6 K/hour. C_12_Cer-1-P was measured in a TA Instruments NANO- DSC with a scan rate of 0.5 K/minute. At least 7 scans of each sample were performed.

### Preparation of large unilamellar vesicles (LUVs)

Mixed lipid solutions in chloroform (SM, cholesterol) or chloroform∶methanol∶water (80∶20∶2 v/v/v, for C_12_Cer-1-P-containing mixtures) were evaporated under a gentle nitrogen stream and kept under vacuum overnight to remove residual solvent. The dry lipid films were hydrated in reaction buffer (PBS, 2 mM MgCl_2_, pH 7.4) preheated to 55°C. The concentrations were adjusted to 0.5 mM for SM. For the enzyme kinetic studies all samples provided equal concentration of substrate (0.25 mM). The samples were vortexed for 90 minutes at 55°C until all lipid was dispersed. The dispersions were freeze-thawed 3 times before extrusion through Whatman 0.1 µm membranes (PC, Track-Etch) using a mini-extruder from Avanti Polar Lipids (Alabaster, AL, USA). Vesicle preparations were extruded 18 times at 55°C. Loss of lipids from extrusion was measured by phosphorus analysis [28], [29] and recovery was always within the error of the technique (±5%). LAURDAN and DiIC_18_ labeling were at 0.25 mol% of total lipids by premixing the probes with the lipids before evaporation. Although pure C_12_Cer-1-P could be suspended in aqueous medium preparation of LUVs using this procedure was unsuccessful.

### Preparation of giant unilamellar vesicles (GUVs)

GUVs were generated using previously described procedures of electroformation in a home-built array of chambers, which contain two Pt wire electrodes each [30]. First, 0.2 mg/ml solutions of lipid (mixtures) in chloroform including the fluorescent probe (0.5 mol% respect to total lipids) were deposited on the electrodes (4 µL per wire). The remaining solvent was evaporated under vacuum for 30 min. For the electroformation of GUVs in salt-free environment, a 2 V, 10-Hz alternating electric field was applied for ca. 90 minutes [31] using a Digimess FG100 function generator (Vann Draper Electronics, Stenson, Derby, UK). This preparation was performed in sucrose osmotically matched to the reaction buffer (either PBS, 2 mM MgCl_2_, pH 7.4, 286 mOsM, or Hepes buffered saline). For electroformation of GUVs under physiological conditions (same buffer supplemented with 50 mM of sucrose), the following protocol was applied: (a) 5 min at 106 mV, 500 Hz; (b) 20 min at 940 mV, 500 Hz; (c) 90 min at 2.61 V, 500 Hz [32], [33]. For electroformation the chambers and solutions were thermostated to temperatures above the main transition temperature of the component with the highest main transition (40°C for C_12_SM, 45°C for the lipid mixtures). Afterwards, the solutions containing GUVs were cooled to room temperature (RT) and removed from the chamber using a pipette. An aliquot of each sample was diluted (1∶4) in the reaction buffer alone (GUVs prepared without salt) or buffer supplemented with 50 mM of glucose (GUVs prepared in physiological conditions), added to an 8 well chamber (μ-slide, Ibidi, Germany) and allowed to settle at RT overnight. After this step the GUVs were studied in the fluorescence microscope. In some of these experiments the GUVs were immobilized as described in [34]. Preparation of GUVs for mixtures of C_12_Cer-1-P/C_12_SM containing 50 mol% or more of C_12_Cer-1-P was unsuccessful using these procedures.

### Recombinant enzymes

Expression and purification of recombinant soluble and active C-terminal His-tagged sphingomyelinase D from *Loxosceles laeta*, isoform 2, was done as previously reported [35]. The inactive isoform Lb3 from *Loxosceles boneti* used as control was His-tagged at the N-terminus and expressed following the same protocol as for the active enzyme. The Lb3 protein was enzymatically inactive. Protein quantitation was done using the Bicinchoninic acid reagent (BCA, Thermoscientific, USA) using the protocols and standards supplied with the reagents. Both proteins were stored at 4°C in stock solutions at 250 µg/ml in PBS buffer. Recombinant enzyme purity was always greater than 95% by SDS-PAGE and protein concentration and activity were assayed at regular intervals.

### Assay of sphingomyelinase activity

Enzymatic activity was measured using a cuvette-based coupled enzymatic assay in the reaction buffer (PBS pH 7.4, 2 mM MgCl_2_) in a 1 ml final volume. The assay follows the oxidation of choline (released by SMD) to betaine by choline oxidase (from *Alcaligenes*, Sigma) and the transformation of the Amplex Red® (Invitrogen) substrate into resorufin by horseradish peroxidase (Sigma) in the presence of hydrogen peroxide released by choline oxidation. Resorufin production was followed spectrophotometrically (Perkin Elmer Lambda 35 UV/VIS spectrometer) by measuring the absorbance at 570 nm. Briefly, known amounts of recombinant SMD (ranging from 0.78 to 50 ng/ml) were incubated with a master mixture such that the final concentration of SM was always 0.25 mM -presented either as mixed micelles (in 0.2% Triton X-100) or LUVs-, 0.1 U/ml choline oxidase, 1 U/ml horseradish peroxidase and 0.05 mM Amplex Red. The specific activities of both choline oxidase and horseradish peroxidase were independently calibrated to ensure quantitative detection of choline production by sphingomyelinase D. Units (U) are always µmoles of resorufin produced (molar extinction of resorufin ε_570_ = 54,000 M^−1^). Specific activities of SMD on various substrates were calculated by dividing the U/min during the linear phase of the reaction by the amount of SMD used in the assays using at least three different SMD concentrations per specific activity measurement. The SMD concentrations were chosen such that the slope of the reactions depended solely on the concentration of SMD. Each kinetic determination was run in duplicate and repeated at least twice. The statistical significance of differences in reaction rates and/or specific activities were established by pairwise comparisons using Student's t-test and globally by ANOVA.

### Dynamic light scattering

Dynamic light scattering is a widely used method for determining an average size or a size distribution of scattering particles in a suspension. Unlabeled C_12_SM, C_12_SM∶C_12_Cer-1-P and C_12_SM∶cholesterol LUVs were prepared as described above. Dynamic light scattering experiments were done in a Brookhaven Instruments goniometer setup (BI-200SM) equipped with a Melles Griot He-Ne laser, λ = 632.8 nm. The measurements were performed at a single scattering angle, 90°, in homodyne detection mode. Cylindrical glass cuvettes, with an outer diameter of 10 mm, were embedded within an index matching and thermostating bath. The glass cuvettes were autoclaved prior to use. The sample temperature was maintained at 25°C during the measurements. The autocorrelation function generated from the intensity fluctuations was analyzed to obtain the distribution of particle sizes using two techniques: multiple pass Non-Negative Least Squares (NNLS) and a regularized NNLS, CONTIN [36].

### Fluorescence spectroscopy measurements

All bulk fluorescence measurements were done in quartz cuvettes in an ISS Chronos spectrofluorometer (ISS, Champaign, USA) at 22°C. LAURDAN Generalized Polarization (GP) was measured using a 374 nm LED excitation source and fluorescence intensity at 440 and 490 nm was collected for GP calculations [37] (see below). To eliminate scatter a long pass filter cutting below 408 nm was located before the emission monochromator. For the Förster Resonance Energy Transfer (FRET) experiments with LAURDAN and DiIC_18_, excitation was as above but emission collected at 590 nm (the emission maximum of DiIC_18_). Protein fluorescence emission spectra (originated mainly by the aromatic amino acids tryptophan and tyrosine) were obtained using a 280 nm diode excitation source. For protein fluorescence polarization measurements, a “L" configuration was used. The excitation polarizer was set at vertical position and the emission polarizer was rotated from vertical to horizontal position using a step-motor. Data were collected at 340 nm for 10 minutes for each sample and the results were expressed as the average of all values ± the averaged standard error of the measurements. For protein fluorescence polarization measurements an additional bandpass filter (350±25 nm) was mounted before the emission monochromator and short path cuvettes (0.4 cm) were used to minimize scatter noise of the samples. The statistical significance of differences were established by pairwise comparisons using Student's t-test.

### Interpretation of the LAURDAN GP function

The LAURDAN GP is a function of the probe's emission spectrum position. The fluorescence emission properties of LAURDAN are sensitive to the water dipolar relaxation processes that occur in the environment of the probe [38]. The energy of the excited state progressively decreases as dipolar relaxation increases, shifting the probe's emission spectrum to longer wavelengths. The extent of water dipolar relaxation observed in highly packed membrane regions (such as the solid-ordered phase in bilayers) is very low compared to what is observed in less packed regions (such as the liquid-disordered phase in bilayers) [37], [39]. Therefore, when a solid-ordered to liquid-disordered phase transition occurs in the membrane, a prominent red shift in the fluorescence emission spectrum of the probe is observed (from blue to green; an almost 50 nm shift). The GP function was defined [39] analogously to the fluorescence polarization function as:
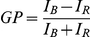
where *I_B_* and *I_R_* correspond to the intensities at the blue and red edges of the emission spectrum (440 and 490 nm) using a given excitation wavelength. In lipid bilayers high LAURDAN GP values (0.5–0.6) correspond to laterally ordered phases (e.g solid-ordered or gel) whereas low LAURDAN GP values (below 0.1) correspond to liquid-disordered phases [37], [39].

### Fluorescence correlation spectroscopy (FCS)

C_12_SM LUVs prepared as described above were labeled with 0.02 mol% of the fluorescent lipid probe Atto647-DPPE. Labeled liposomes were separated from potentially free fluorophores by chromatography through a Sephacryl S400 column. For the FCS measurements, labeled LUVs were diluted 1∶100 in unlabeled C_12_SM LUVs (final concentration 0.25 mM C_12_SM). The measurements were carried out by means of a custom built multiphoton excitation system constructed in a Nikon Eclipse TI microscope. The objective used was a 60× water immersion objective, NA 1.29. The excitation light source was a femtosecond Ti∶Sa laser (HPe Mai Tai DeepSee, tunable excitation range 690–1040 nm, Spectra Physics, Mountain View, CA) and the excitation wavelength was 838 nm. The fluorescence signal was collected using a bandpass BrightLine HC 676/29 nm. All filters were from AHF Analysetechnik, Germany. The fluorescence was detected by means of a Hamamatsu H7422P-40 photomultiplier. The microscope is controlled by SimFCS (Laboratory for Fluorescence Dynamics, University of California, Irvine, CA, USA). The FCS data was analyzed using SimFCS and Globals for Spectroscopy (Laboratory for Fluorescence Dynamics, University of California, Irvine, CA, USA) using the model of single or two species diffusing in 3D Gaussian 2-photon excitation volume detailed in [40], [41]. Particularly, the data generated from the C_12_SM liposomes was fitted using the model above for two diffusing species in 3D. Two diffusion coefficients (D_1_ and D_2_) were used for analysis for two reasons: the first is that although the initial state corresponds to a largely homogeneous LUV preparation, there are however small heterogeneities present; the second is that, after enzyme addition, not all vesicles would be expected to be affected in the same way and in the same time window. Therefore, for the analysis of the enzymatic effects D_1_ was fixed and the program allowed generating the best fit by optimizing the value of the second diffusion cofficient D_2_. Diffusion coefficients were used to calculate particle radii using the Stokes-Einstein relationship [42]. The data for the FCS measurements was typically collected for 3 min. The experiments were carried out at 21°C.

### Fluorescence microscopy

Fluorescence imaging of DiIC_18_-labeled GUV preparations was carried out in an inverted, confocal laser scanning microscope (Zeiss LSM 510 META NLO, Carl Zeiss, Jena, Germany) at room temperature. Both 40× and 63× water-immersion objectives with a NA of 1.2 were used. DiIC_18_ was excited at 543 nm, and its fluorescence signal was collected applying a 560 nm long-pass filter. The images are contrast-enhanced and the background was cut-off by image-processing software. LAURDAN GP in GUVs was measured in the same custom-built, two-photon excitation microscope used for FCS measurements with a 60× NA 1.2 water-immersion objective. At least 20 individual vesicles were analyzed from at least two independent preparations. LAURDAN was excited in the two-photon excitation mode at 780 nm. The fluorescent signals of the probe's emission spectrum were split with a dichroic mirror centered at 470 nm and collected using band-pass filters of 438±12 nm and 494±10 nm simultaneously with two Hamamatsu H7422P-40 photomultipliers. Based on the acquired intensity images, LAURDAN GP images were computed with SimFCS software (Laboratory for Fluorescence Dynamics, Irvine, CA). The GP images were calibrated with a correction factor G obtained from a LAURDAN GP standard (2 µM LAURDAN in DMSO) as previously described [43]. The LAURDAN GP value of the reference solution was measured in an ISS Chronos spectrofluorometer (ISS, Champaign, USA) as described above.

## Results

### Effects of Cer-1-P in pre-mixed SM model membranes

**Figure 1:**
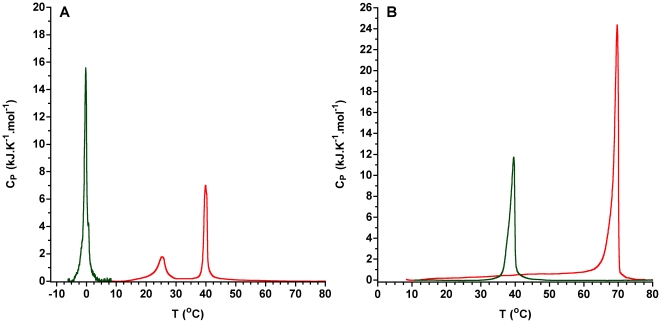
Differential scanning calorimetry of pure sphingomyelins and ceramide-1-phosphates. Thermogram of A) C_12_SM (green) and C_12_Cer-1-P (red). B) Thermogram of egg SM (green) and and C_16_Cer-1-P (red).

Differential scanning calorimetry experiments show that Cer-1-P exhibits a much higher main transition temperature than the parent SM (Fig. 1). In addition, C_12_Cer1P dispersions show two endotherms but only one major change around 40°C (from 0.597±0.005 at 22°C/30°C to −0.027±8×10^−6^ at 50°C) occurs in the value of the LAURDAN GP similar to that observed at the main phase transition temperature of pure glycerophospholipids [39].

**Figure 2:**
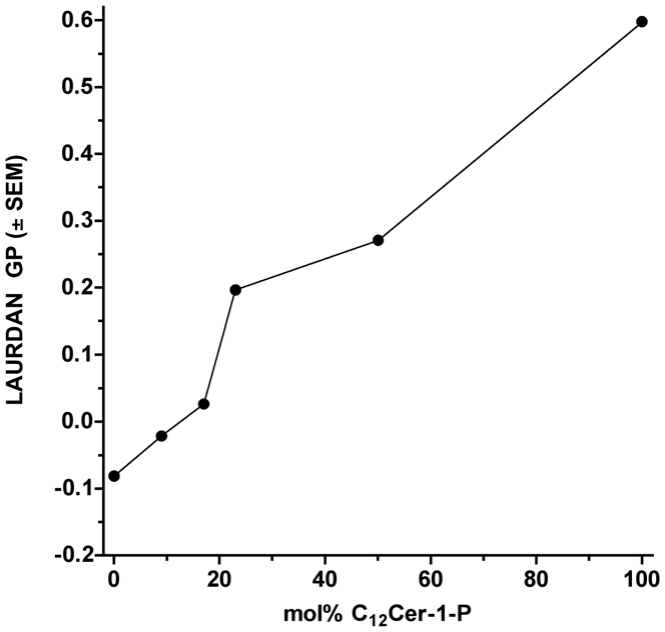
LAURDAN GP of C_12_SM∶C_12_Cer-1-P mixtures at 22°C. The LAURDAN GP of suspensions of mixed C_12_SM and C_12_Cer-1-P at different ratios was measured. Pure C_12_Cer-1-P could not be extruded so the LAURDAN GP measurement was taken directly on the lipid suspension.

Premixing of C_12_SM with increasing amounts of C_12_Cer-1-P decreases the extent of dipolar relaxation in the membranes as measured by the polarity sensitive LAURDAN probe in lipid suspensions. LAURDAN GP measurements at 22°C, that is, a temperature between the main transitions of both lipids, show a monotonic increase from values corresponding to a liquid disordered (*l_d_*) phase to a more highly packed structure (Fig. 2). In particular, pure C_12_Cer-1-P dispersions display a GP value of 0.6 indicating a low degree of solvent dipolar relaxation. In glycerophospholipids this value corresponds to a solid ordered phase (*s_o_*), the so-called gel phase [39].

**Figure 3:**
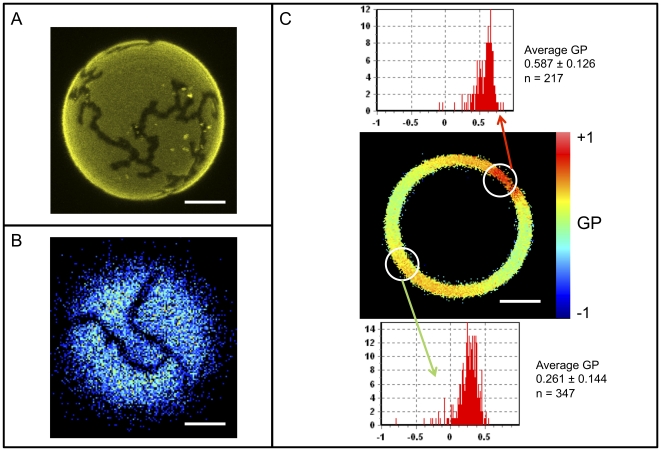
Pre-mixed C_12_SM∶C_12_Cer-1-P domains in GUVs. Representative images of domains in individual vesicles composed of 23 mol% C_12_Cer-1-P visualized with (A) DiIC_18_ which is excluded from the C_12_Cer-1-P-enriched domains, B) LAURDAN intensity image at the pole of a giant vesicle where photoselection prevents probe excitation in the more ordered domains and, C) LAURDAN GP analysis of domains in an equatorial section showing areas of low dipolar relaxation (high GP, top histogram) and higher dipolar relaxation (lower GP, bottom histogram). Histograms were determined opposite to each other to avoid any bias caused by photoselection. Bars are 5 µm.

Examination of this system by fluorescence microscopy using DiIC_18_ and LAURDAN provides spatially-resolved data for interpretation of the behavior of these lipid membranes. When GUVs are prepared from premixed C_12_SM and C_12_Cer-1-P and examined microscopically at room temperature, the formation of distinct domains can be visualized by the exclusion of DiIC_18_ (Fig. 3A). These Cer-1-P-enriched domains are more tightly packed as revealed by the well known photoselection effect of LAURDAN [44] observed in the intensity images obtained at the GUV's polar region (Fig. 3B) and the local LAURDAN GP imaging measurements (Fig. 3C) obtained at the equatorial region of the vesicle. This shows that at 22°C the C_12_SM∶C_12_Cer-1-P mixture exhibits *s_o_/l_d_* phase coexistence, as reported for binary mixtures of glycerophospholipids [45], [46] and a POPC∶C_16_Cer-1-P mixture at X_Cer-1-P_>0.3 [47].

### Kinetics of SMD activity on C_12_SM vesicles

**Figure 4:**
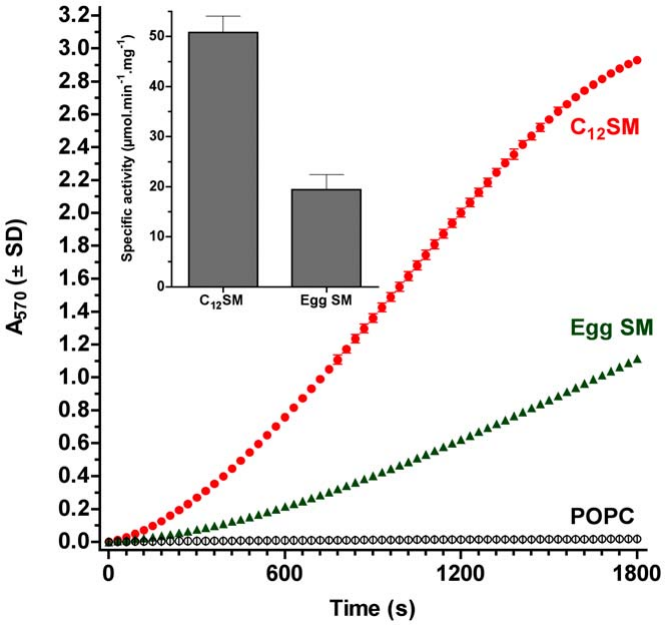
Kinetics of SMD activity on large unilamellar vesicles. The main panel shows a representative trace of the kinetics of SMD activity on LUV preparations of C_12_SM (red), egg SM (green) and POPC (black) at 22°C. Inset: Specific activity of SMD on C_12_SM and egg SM LUVs. The difference in specific activity is statistically significant (P<0.0001).

The activity of SMD has been often studied using SM as part of uncharacterized lipid dispersions [7]. In our experiments with mixed Triton X-100 micelles, the specific activity of the enzyme is maximal and essentially identical between two SM substrates (lauroyl and palmitoyl SM, 119.8±3.31 and 128.5±13.16 µmol/min per mg SMD, respectively). When we studied these substrates as part of bilayers, however, the activity of the enzyme is significantly lower for both substrates. Interestingly, when the substrate is organized in bilayers, enzyme activity is significantly different between them, with egg SM (initially in the *s_o_* phase at the assay temperature) being processed with 60% lower velocity than the C_12_SM (initially in the *l_d_* phase) (Fig. 4).

**Figure 5:**
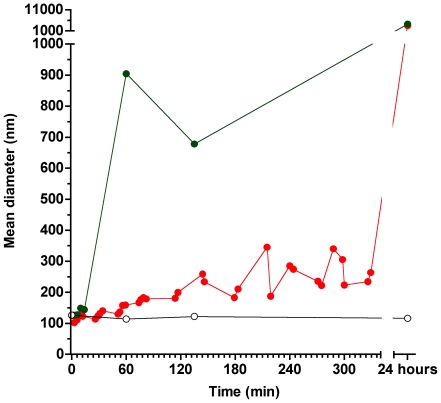
Dynamic Light Scattering of C_12_SM LUVs under the action of SMD. Representative evolution of the size (as mean diameter in nm) of C_12_SM vesicles treated with 50 ng/ml (red) and 1 µg/ml (green) SMD. The open black circles show the scattering behavior of untreated vesicles, which was similar to control POPC vesicles treated with SMD and C_12_SM vesicles treated with inactive Lb3 (not shown).

In order to explore the structural consequences of *in situ* generation of Cer-1-P in SM containing membranes we concentrated on C_12_SM. Dynamic light scattering (DLS) experiments using pure C_12_SM LUV preparations show that extruded vesicles cluster around the expected size of ∼100 nm, and that this size distribution is stable for at least 24 hours in the absence of enzyme (Fig. 5). Addition of SMD causes a progressive increase in sample polydispersity whose time course depends on the amount of enzyme added; 50 ng/ml SMD causes a progressive slow increase in scattering whereas when 1 µg/ml enzyme is added, scattering exceeds the limits of meaningful size distribution analysis within an hour. The increase in polydispersity was apparent when comparing two different methods of computation, non-negative least squares (NNLS) and CONTIN, which showed a few punctual differences but overall agreement (P<0.001).

**Figure 6:**
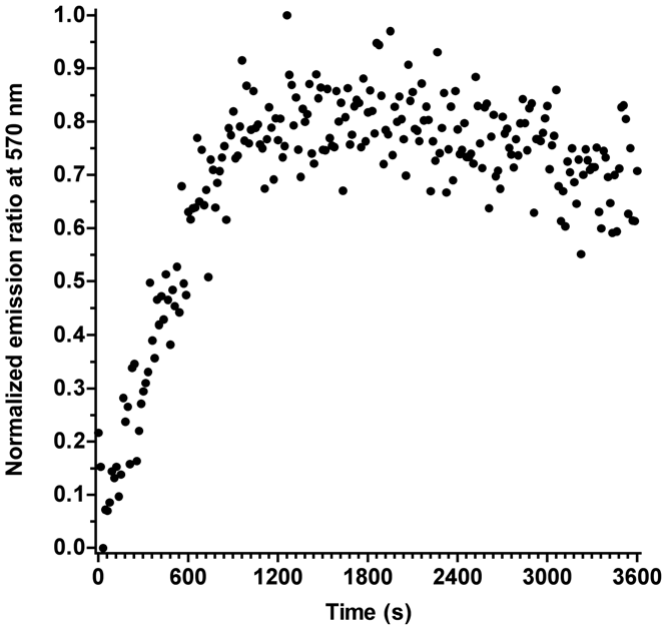
Förster resonance energy transfer (FRET) of LAURDAN- and DiIC_18_-labeled LUVs under the action of SMD. Separately labeled vesicles were mixed at a 1∶1 ratio and treated with SMD (1 µg/ml). Emission at the DiIC_18_ maximum (with excitation at 374 nm) was followed in time relative to a reference of an identical liposome mixture without enzyme. The ordinate is the normalized ratio of fluorescent emission of the treated over the untreated control.

To independently explore the mechanism underlying the DLS data, we conducted a series of fluorescence experiments. The first one was a simple assay with unilamellar vesicles independently labeled with two membrane fluorophores that function as a FRET pair. LUVs separately labeled with LAURDAN and DiIC_18_ were incubated with SMD, excited at 374 nm and the DiIC_18_ emission followed. As shown in Fig. 6, there is a marked increase in the DiIC_18_ emission at 590 nm over the untreated mixture of vesicles, indicating an increase in donor-acceptor interaction.

**Figure 7:**
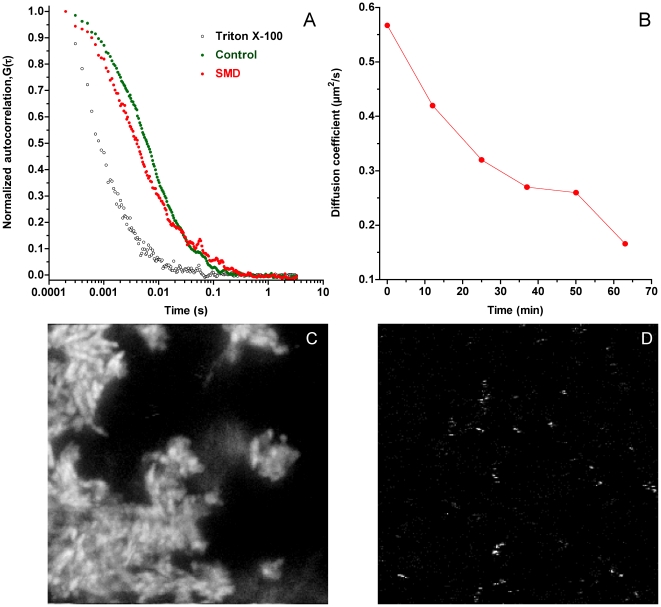
Fluorescence correlation spectroscopy of C_12_SM LUVs under the action of SMD. A) Representative normalized autocorrelation plots for the untreated vesicles (green circles), the same preparation treated with 1 µg/ml SMD (red circles) and a control with Triton X-100 (open black circles). B) Diffusion coefficient (D_2_) in time calculated from fitting the autocorrelation plot for the enzyme-treated sample. The bottom panels represent fluorescence images taken at 24 hours of C) sedimented material after enzyme treatment (1 µg/ml SMD) and D) untreated C_12_SM LUVs. The images are 30 µm×30 µm.

**Figure 8:**
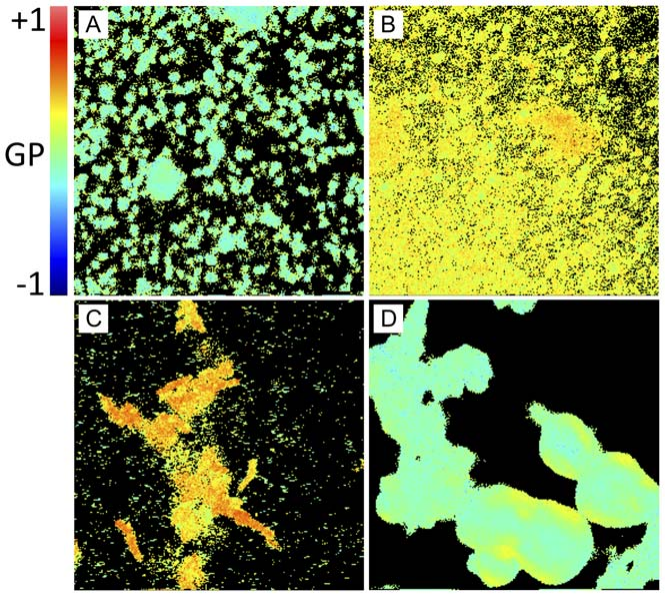
LAURDAN GP image of sedimented material from C_12_SM LUVs treated with SMD. A) Sedimented control 100 nm liposomes after 24 hours (without SMD) at the bottom of the slide. Average GP value is −0.029±0.129. B) Sedimented material of LUVs treated with 1 µg/ml SMD after 24 hours at the bottom of the slide. Average GP value is 0.228±0.088. C) Same as B) but 5 µm above the slide surface. Average GP of 0.269±0.176 with areas of GP of 0.452±0.124. D) Sedimented material from the 50 mol% pre-mixed lipids without enzyme. Average GP of −0.044±0.079. Fields are 19 µm×19 µm.

The second experiment consisted of measurements by fluorescence correlation spectroscopy (FCS) of C_12_SM LUVs labeled with Atto 647-DPPE (approx. 20 molecules per vesicle) mixed with unlabeled vesicles (cf. Methods). The resulting autocorrelation curves of the LUV preparation could be fitted by a single diffusion coefficient, however, a two diffusion component fit proved more adequate for the data (typical curves are shown in figure 7A). One of the component's diffusion coefficients (D_1_) was found to be 4.6±0.25 µm^2^/s corresponding to a radius of about 44 nm, in agreement with the DLS measurements of C_12_SM LUVs. Addition of the enzyme to the C_12_SM vesicles had two clear consequences. First a significant decrease in labeled particle mobility was observed, i.e. a time-dependent decrease in the calculated diffusion coefficient (D_2_) of the second component of the fit (Fig. 7B). This effect is interpreted as an increase in the particle size. Second, an important redistribution of the fluorescent probe became apparent. This is seen as a decrease in the average intensity of the individual diffusing particles measured in the bulk of the suspension, with a concomitant sedimentation of bright microscopic structures to the bottom of the chamber (Figure 8C). Surprisingly, the number of fast diffusing particles (those contributing to D_1_) was not seen to decrease (as indicated by a decrease of G_0_ of the autocorrelation curves, which is inversely proportional to the number of fluorophores in the observation volume). We interpret this as a consequence of fusion of labeled and unlabelled vesicles causing dilution of the probe (see Methods). This effect is not seen in the control measurements without enzyme. As an additional control measurement, an equal volume of 0.2% Triton-X100 was added to the mixture of unlabelled and labelled C_12_SM LUVs that resulted, as expected, in a homogenous particle distribution (that could be fitted by a single component) with a faster diffusion coefficient of 27±3 µm^2^/s corresponding to a mean micelle radius of ∼7.8 nm.

Interestingly, large deposits of non-diffusing labeled material could be visualized by microscopic inspection of the FCS field of view after 24 hours of incubation of C_12_SM LUVs with SMD (Fig. 7C). These structures are not apparent in the untreated sample (Fig. 7D), which continues to reveal quickly diffusing particles. A LAURDAN GP image of the sedimented material reveals that these structures are largely different both in shape and in GP values (Figs. 8 B and C) from the untreated control (Fig. 8A) and a C_12_SM and C_12_Cer-1-P (Fig. 8D) mixture in the absence of enzyme.

**Figure 9:**
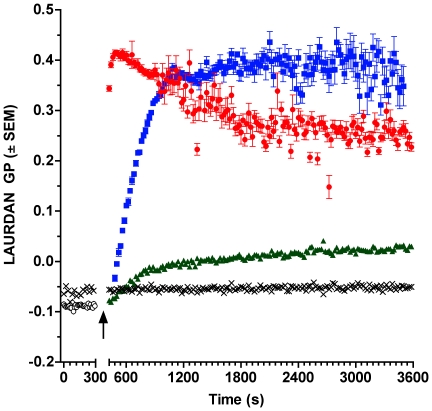
Representative kinetics of LAURDAN GP of C_12_SM LUVs treated with SMD. LAURDAN-labeled C_12_SM LUVs were treated with different concentrations of SMD: 100 ng/ml (green triangles), 1 µg/ml (blue squares) and 2.5 µg/ml (red circles). The black open circles are the same vesicles before addition of enzyme and the black crosses are non-substrate POPC LUVs with 2.5 µg/ml SMD. The arrow indicates the time of SMD addition.

When pure C_12_SM LUVs labeled with LAURDAN are followed in time during enzymatic action, LAURDAN GP increases progressively (Fig. 9), consistently with the GP measurements obtained with the increasing molar fraction of C_12_Cer-1-P in the premixed C_12_SM∶C_12_Cer-1-P vesicles (cf. Figs. 2, 3B and 3C). At different enzyme concentrations, however, the evolution of the GP follows very different kinetics in bulk measurements. At high enzyme concentrations (1 and 2.5 µg/ml) the GP reaches a maximum. This state evolves and slowly decays at 2.5 µg/ml whereas it remains slightly more stable at 1 µg/ml although the noise of the readings noticeably increases. When a lower enzyme concentration is used the overall GP does not reach this maximum reflecting a lower degree of hydrolysis in the time window of the experiments.

**Figure 10:**
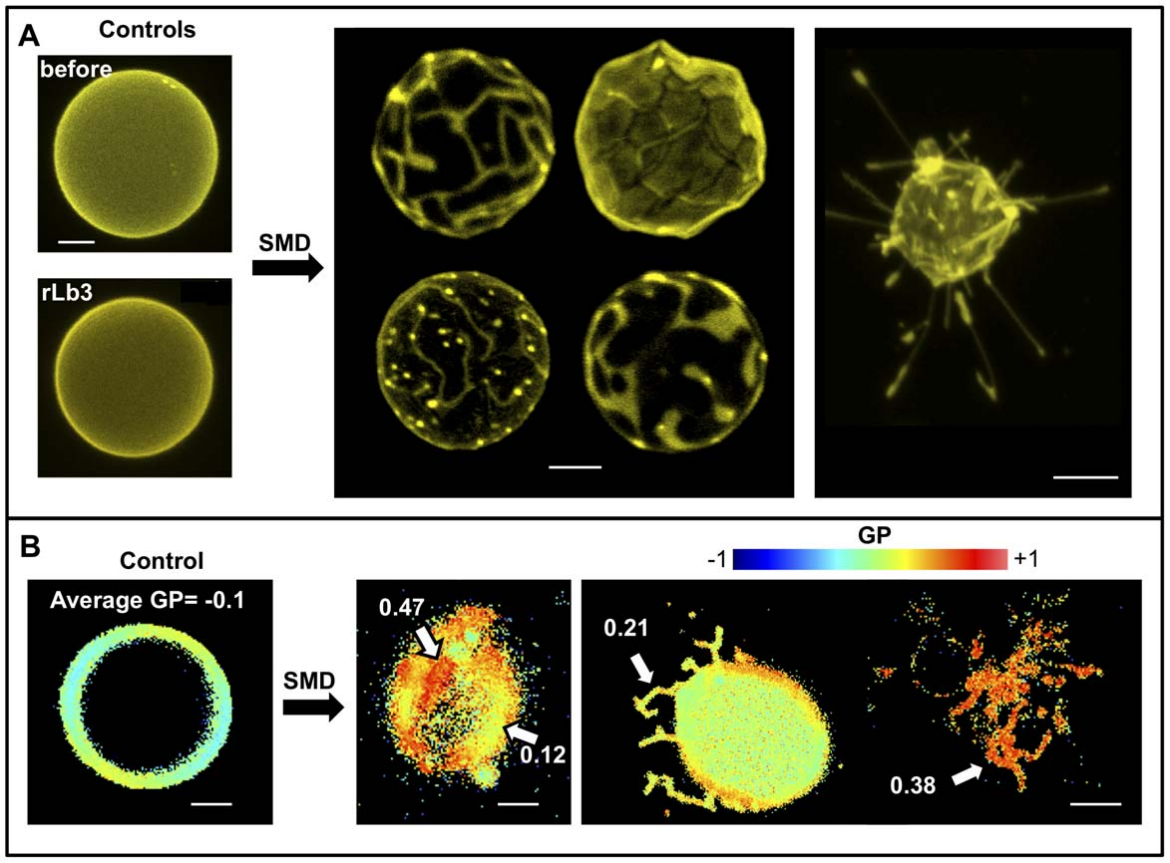
Representative fluorescence microscopy images of the action of SMD on C_12_SM giant unilamellar vesicles. A) DiIC_18_-labeled GUVs. Top left shows a typical vesicle before SMD addition and bottom left a GUV after 17 hours of incubation with inactive Lb3. The center panel shows domain formation in several GUVs 1–3 hours after SMD addition. On the right a collapsed vesicle with extruded tubes. B) Changes in LAURDAN GP induced by SMD. The left panel shows an untreated GUV, homogeneously fluid. The center panel shows domains of different GP value (indicated by arrows). The right panel shows a GUV with tubular extrusions (left) and a collapsed one (right). Bars are 5 µm.

In order to obtain spatially resolved information on the effects of SMD action on membranes, we performed fluorescence microscopy experiments using GUVs. The general observation is that morphologically distinctive structures appear in GUVs upon enzymatic action: many vesicles collapse, some vesicles exhibit coexisting domains and others extrude tube-like projections. The presence of domains can be visualized by DiIC_18_ exclusion (Fig. 10A central panel) and LAURDAN GP measurements (Fig. 10B central panel). LAURDAN GP images as well as the observed photoselection effect of the probe show that the areas excluding DiIC_18_ exhibit significantly less dipolar relaxation (more *s_o_*-like character) than the surrounding membranes ((*l_d_* -like character). Additionally, the tubular structures that can be observed by DiIC_18_ (Fig. 10A right panel) were analyzed by measurement of the LAURDAN GP. The GP data indicate that dipolar relaxation in these projections is lower than the control GUVs (cf. Fig. 10B right and left panels).

### The interaction between SMD and C_12_SM membranes

In interfacial catalysis two alternative models of interaction of enzymes with organized substrates (membranes) have been proposed [48]. The differences hinge upon whether the enzyme remains adsorbed onto the supramolecular structure formed by the substrate between catalytic events (“scooting") or whether it exchanges between the substrate and the surrounding aqueous medium between catalytic events (“hopping"). To explore whether the enzyme operates under the scooting or hopping modes we performed kinetic studies [49] under conditions in which the velocity of the enzyme is governed by the concentration of C_12_SM present: 0.05 and 0.025 mM, which give linear slopes (Absorbance/s) of 4.97×10^−4^ and 1.86×10^−4^, respectively. Increasing the concentration of substrate during the linear phase of the reaction from 0.025 to 0.05 mM caused only a modest increase in reaction velocity, from 1.86×10^−4^ to 2.22×10^−4^ OD/s (a 16.7% increase). The final velocity remained well below the velocity of the reaction measured at the higher substrate concentration, indicating that a significant part of the enzyme does not readily exchange with freshly added vesicles. This suggests that there is an important component of “scooting" in SMD action.

**Figure 11:**
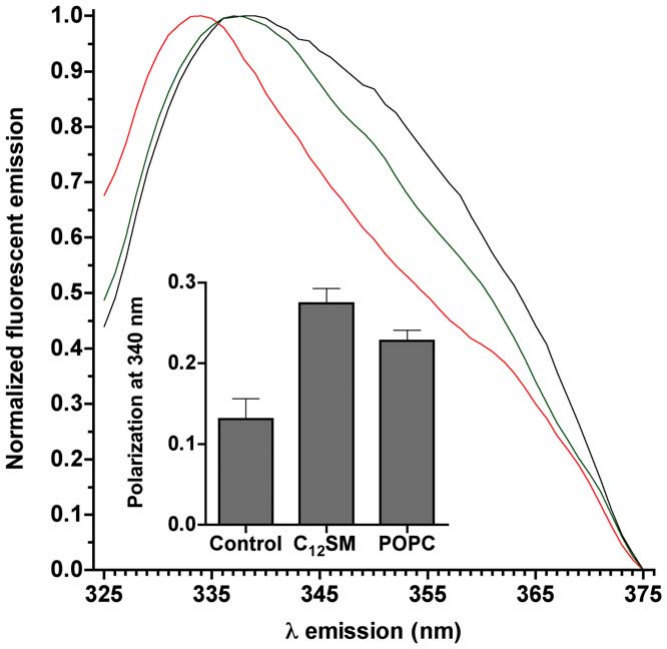
Protein fluorescence spectra of SMD. The main panel shows the normalized fluorescence emission spectrum of SMD (excitation at 280 nm). The black trace is SMD alone, mixed with POPC vesicles (green) and with C_12_SM LUVs (red). Inset: fluorescence polarization values for the same samples. The differences in polarization are statistically significant (P<0.003).

Protein fluorescence polarization and emission spectral shift experiments also indicate an important degree of association between enzyme and vesicles. Addition of LUVs to an enzyme solution results in a marked blue shift of the protein emission spectrum (Fig. 11), which depends on the composition of the membranes: while the emission spectrum of SMD is slightly shifted by non-substrate POPC vesicles with respect to the protein in solution, the shift is much more pronounced in the presence of C_12_SM membranes. A significant degree of association between the enzyme and C_12_SM membranes is also apparent when fluorescence polarization values of SMD alone or in the presence of membranes are compared (Fig. 11 inset).

### Effect of product on reaction rates

**Figure 12:**
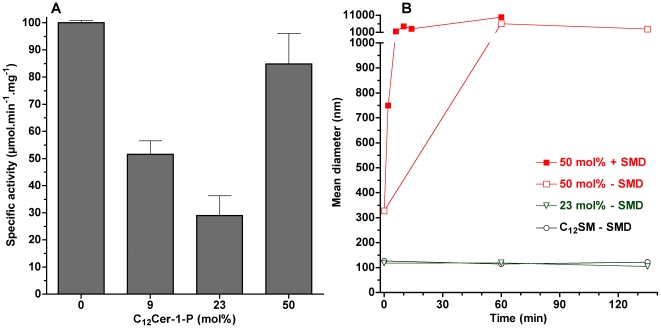
Effect of product on relative enzyme specific activity and on vesicle size. A) Specific activity of SMD on C_12_SM LUVs with increasing molar fractions of C_12_Cer-1-P. With the exception of pure C_12_SM and the 50 mol% mixture, all other differences are statistically significant (P<0.003). B) Mean diameter in nm versus time obtained by DLS of C_12_SM∶C_12_Cer-1-P LUVs: The black open circles represent pure C_12_SM untreated vesicles, the green triangles represent untreated C_12_SM∶C_12_Cer-1-P vesicles (23 mol%), the closed red squares are C_12_SM∶C_12_Cer-1-P vesicles (50 mol%) treated with 1 µg/ml SMD and the open red squares are the same but without enzyme.

When C_12_SM is pre-mixed with the product at different molar ratios and subjected to SMD, the velocity of the enzymatic reaction is sensitive to the proportion of C_12_ Cer-1-P present. The effect is not linear: using LUVs at mol fractions of 9 and 23 mol% of Cer-1-P enzymatic activity is significantly lower than for pure C_12_SM. However, at a molar ratio of 50%, enzyme activity is comparable to pure C_12_SM (figure 12A).

To further explore this unexpected increase in reaction rate, we decided to further explore the behavior of LUVs of these compositions using DLS in the absence of enzyme. Vesicles containing up to 23 mol% C_12_Cer-1-P displayed a stable scatter of light for up to 24 hours. However, LUVs composed of 50 mol% mixture of C_12_SM∶C_12_Cer-1-P were unstable in the sense that scattering increased at a rate roughly comparable to that of the samples exposed to the enzyme (Fig. 12B). Additionally, this mixture shows greater scatter at time 0 compared to the other samples indicating spontaneous formation of different structures shortly after extrusion. These observations preclude a direct comparison between reaction rates in this mixture and the other (initially stable) lipid compositions.

### Effect of cholesterol

**Figure 13:**
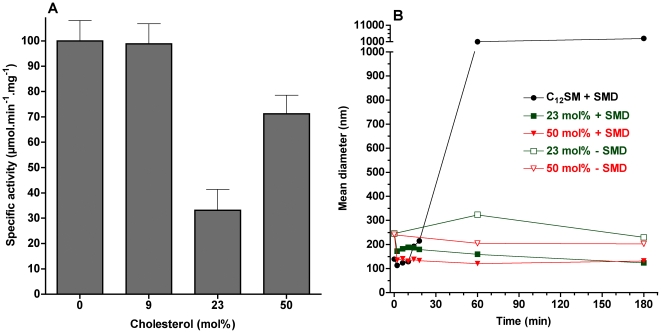
Effect of cholesterol on relative enzyme specific activity and on vesicle size. A) Effect of cholesterol on the specific activity of SMD. With the exception of pure C_12_SM and the 9 mol% mixture, all other differences are statistically significant (P<0.003). B) Mean diameter in nm versus time obtained by DLS of C_12_SM∶Cholesterol LUVs: The black circles represent pure C_12_SM vesicles with SMD, in green C_12_SM∶cholesterol vesicles (23 mol%) with (closed squares) and without (open squares) SMD and in red C_12_SM∶cholesterol vesicles (50 mol%) with SMD (closed triangles) and without (open triangles). Enzyme concentration was 1 µg/ml.

Vesicles prepared from C_12_SM with increasing molar fractions of cholesterol show an increase in the LAURDAN GP values expected for a gradual formation of a liquid ordered (*l_o_*) phase (0.080±0.002 at 9 mol%, 0.452±0.019 at 23 mol% and 0.555±0.017 at 50 mol%) in agreement with previous reports [50]. At a low cholesterol level (9 mol%) enzyme activity is mostly unaffected whereas at 23 mol% a minimum of activity (32%) is reached. Raising cholesterol to the maximum possible incorporation increases the specific activity of the enzyme but without reaching the value of pure C_12_SM (Fig. 13A). When examined in terms of light scattering behavior (Fig. 13B), SMD action on C_12_SM∶cholesterol vesicles did not increase polydispersity and, in fact, vesicle size slightly decreased relative to controls without enzyme. This effect was more pronounced in the 50 mol% mixture. In the absence of enzyme the size distribution of vesicles remains stable in the time window of the experiment.

**Figure 14:**
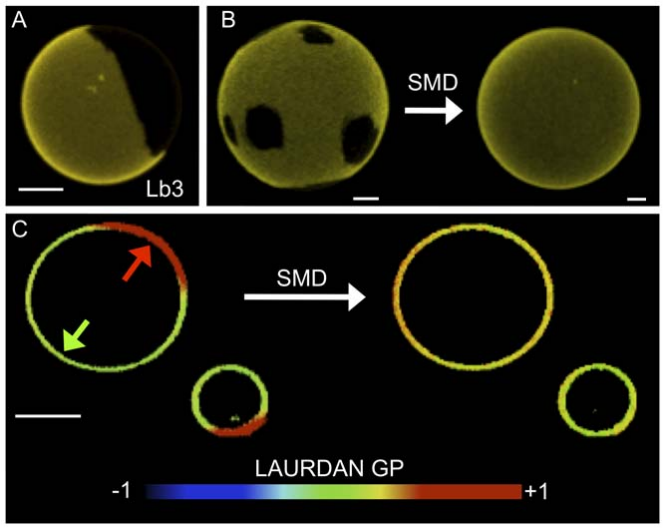
Representative fluorescence images of the effect of SMD on GUVs composed of DOPC∶eggSM∶cholesterol 2∶1∶1 mol. A) Domains in a DiIC_18_-labeled GUV exposed to inactive Lb3 for 16 hours. B) Representative DiIC_18_-labeled GUVs before and after treatment with SMD. C) Two LAURDAN-labeled GUVs before (left) and after (right) exposure to SMD. Before treatment two domains are apparent (average GP values of 0.1 and 0.5 indicated by the green and red arrows, respectively), after SMD action the membrane becomes uniform (average GP value of 0.3). Bars are 5 µm.

When a more complex ternary mixture of lipids composed of DOPC∶egg SM∶cholesterol (2∶1∶1 molar ratio) is treated with SMD an interesting effect is observed. Initially, these GUVs exhibit domain coexistence as visualized by DiIC_18_ images (Fig. 14A and left vesicle in Fig. 14B) and LAURDAN GP measurements (Fig. 14C, left), in agreement with previously reported data for this lipid mixture [51]. The action of SMD on these vesicles results in the disappearance of the micron-sized domains evident in DiIC_18_ images (Fig. 14B, right) and LAURDAN GP measurements (Fig. 14C, right). Enzymatic action results in an intermediate GP value 8 hours after enzyme addition. Domains were unaffected in the absence of active enzyme (buffer alone, not shown) or with the same amount of inactive Lb3; Fig. 14A).

## Discussion

### Methodological considerations and characterization of mixed substrate-product membranes without enzyme

Although we performed some experiments using egg SM and C_16_Cer-1-P, we chose to study the effects of enzymatic conversion in model membranes where C_12_SM is transformed into C_12_Cer-1-P. Among the reasons for this choice are: i) the compositional simplicity of the system, ii) that at room temperature the membranes composed of mixtures of the substrate (C_12_SM) and the product (Cer-1-P) can show *l_d_*/*s_o_* phase coexistence and, iii) the enzyme is active at this temperature. This model may approximate some aspects of more complex membranes, where the action of SMD on sphingomyelins may result in the production of species with much higher melting temperature, and therefore likely to induce the formation of more tightly packed domains than the substrate sphingomyelin. Since C_16_Cer-1-P [27] and egg SM are in the *s_o_* phase at room temperature, their interest as a model for the study of distinguishable phase differences is limited. In any case, our experiments with C_16_Cer-1-P are in qualitative agreement with previous results [27] although the transition temperature reported is slightly lower than the one determined by us. This may be explained in part by the different sources of the lipid used.

Pure C_12_Cer-1-P exhibits two endothermic transitions apparent even after seven scans, unlike C_16_Cer-1-P which shows only one stable transition. For C_12_Cer-1-P, LAURDAN GP measurements above and below the lower endotherm show a persistent high GP value. However, a significant decrease in GP, comparable to that observed in *s_o_* to *l_d_* phase transitions in glycerophospholipids, is only apparent above the higher temperature endotherm. Interestingly, while suspending C_12_Cer-1-P was not difficult it was not possible to prepare LUVs by extrusion or GUVs by electroformation, suggesting that the pure component tends to exist in multiple structures besides the bilayer.

Several observations also reveal structural heterogeneity in the C_12_SM∶C_12_Cer-1-P mixture at high C_12_Cer-1-P molar ratios: i) when mixed with C_12_SM, C_12_Cer-1-P can be incorporated into stable vesicles up to a certain relative concentration which, under bulk equilibrium conditions, would be above 23 mol%; ii) GUVs cannot be successfully prepared at or above 50 mol% composition; iii) the LAURDAN GP of the sedimented material from the 50 mol% mixture shows an average GP that corresponds to pure C_12_SM; iv) when the 50 mol% C_12_SM∶C_12_Cer-1-P mixture is used as the substrate in the enzyme kinetic studies, the reaction rates recover to a level comparable to that of pure C_12_SM. Our interpretation is that there is a limit to the amount of C_12_Cer-1-P that mixed C_12_SM∶C_12_Cer-1-P lamellar structures can accommodate. This stands in contrast to the ability of pure C_16_Cer-1-P, a more symmetrical molecule, to form lamellar structures [27].

C_12_SM is a neutral lipid whereas the product of SMD action is negatively charged. The 50 Å^2^ molecular area of C_12_SM measured by us in Langmuir films at 30 mN/m (corresponding to the pressure equivalence for a bilayer [43], [52]) is greater than the 38 Å^2^ of C_12_Cer-1-P, significantly altering the critical packing parameter. This difference is solely ascribable to the different polar head groups as the hydrophobic moiety remains the same. These different lipid geometries and overall charges may be responsible for a gradual transformation of membrane structure in a C_12_Cer-1-P concentration-dependent manner, affecting membrane curvature and driving the apparent increase in the particle size distribution observed for the 50 mol% mixture.

### Effect of membrane lateral structure on the kinetics of SMD activity

When equal concentrations of C_12_SM and egg SM are presented to the enzyme as bilayers there is a significant difference in activity between both sphingomyelins (∼60% lower for egg SM than C_12_SM). At the temperature of the enzyme activity measurements these two membranes are initially in different phase states; C_12_SM is above its main phase transition temperature (*l_d_*) whereas egg SM is well below its own transition temperature (and therefore in an *s_o_* phase). However, in our experiments using mixed Triton X-100 micelles, C_12_SM and egg SM are processed with essentially identical velocity (119.8±3.3 and 128.5±13.2 µmol.min^−1^.mg^−1^, respectively), indicating that in these conditions the chemical structure of the substrate does not affect enzymatic catalysis. Therefore, the difference in membrane lateral arrangement must underlie the difference in the activity of the enzyme. This influence is additionally supported by the effect of cholesterol, which promotes the formation of a phase with intermediate properties (the so-called liquid ordered [*l_o_*] phase) which also significantly affects enzyme activity in unilamellar vesicles.

SMD action has been studied using a number of lipid species (lysophospholipds, sphingomyelin), but the effect of lipid lateral organization on SMD activity has not been systematically addressed to date. For example, in a study of SMD substrate specificity, enzymatic activity was compared between SM and a variety of lysoglycerophospholipids [7]. It is unclear from this study whether these very different substrate molecules exist as monomers or how they are supramolecularly arranged (e.g. in micelles), and the study assumes that bovine serum albumin “presents" the lipids to the enzyme. In our study, the only protein present in the reactions was the enzyme itself and in these conditions it can also hydrolyze the lysophospholipid C_12_LPC at a concentration slightly above the critical micelle concentration (CMC) with a velocity of 30% that of C_12_SM LUVs (data not shown). Our observations prove that the type of supramolecular arrangement affects SMD activity. This supramolecular organization, in turn, is governed by the chemical structure of the individual sphingomyelin molecules although the chemical structure by itself does not affect enzymatic activity (as the mixed micelle kinetics show). This line of thinking may contribute to a greater understanding of a longstanding question regarding the toxic effects of the ubiquitous venom phospholipases, namely, why are the effects of some phospholipases specific (myotoxic, hemorrhagic, neurotoxic, etc.) when almost all active phospholipases can hydrolyze a wide array of phospholipids *in vitro* and their potential substrates are almost universally present on the surface of cells. Current thinking on the matter emphasizes the role of protein determinants as the source of particular pathophysiological effects [53]–[55]. The supramolecular properties of the target membranes are not generally considered although there is evidence that they impinge on the kinetics of enzymatic activity in model systems [56], [57] and cells [58]. In the case of SMD, it would be of interest to compare different active recombinant proteins in terms of sensitivity to phase state and other parameters of the membranes containing the target lipids. This approach may uncover functional differences of relevance to our understanding of their toxicity and, possibly, evolution [59].

### The effect of enzyme activity on the structure of C_12_SM membranes

Our experiments in LUVs reveal that SMD has several effects on C_12_SM membranes at room temperature. First, there is a marked increase in the LAURDAN GP values during enzyme action, indicating that the amount of C_12_Cer-1-P increases with time. The evolution of this increment depends on enzyme concentration and is exclusive to sphingomyelin containing membranes (Fig. 9). Secondly, enzymatic activity causes a marked increase in the mean size of the lipid structures, observed by DLS and FCS. We interpret this to be the consequence, at least in part, of membrane fusion events as revealed by probe redistribution and supported by donor-acceptor interaction in the FRET experiments. In other words, the enzyme destabilizes an initially uniform population of LUVs and results in an increasingly more complex system. Destabilization was also observed when premixing C_12_SM with a high molar ratio of C_12_Cer-1-P in the absence of enzyme as discussed above. However, the LAURDAN GP images of the sedimented material obtained by premixing the substrate and the product and the mixture generated by enzyme action are different. This suggests that the factors that determine changes in membrane structure are different under equilibrium (premixed substrate and product) and non-equilibrium (enzymatic hydrolysis) conditions. Pre-mixing will generate stable mixed bilayers in both membrane leaflets up to at least 23 mol% C_12_Cer-1-P. The enzyme will, however, initially operate on the available outer leaflet only. Product formation, given the very different molecular characteristics (shape, charge) of the substrate and the product, probably results in the generation of leaflets with different spontaneous curvature thus destabilizing the bilayer and giving rise to the multiplicity of observed structures [60]. This entails that the evolution of the system under enzyme action cannot be fully modeled by pre-mixing progressively greater amounts of product with the substrate (compare Figs. 3 and 10, see above).

In bulk experiments only average quantities can be measured. This limits interpretation of the data because information on local structural changes induced by enzymatic change of lipid composition is missing. This limitation is overcome by spatially resolved measurements using fluorescence microscopy of fluorescently labeled GUVs. These experiments reveal that the heterogeneity inferred from the bulk measurements is a consequence of membrane morphological changes. *In situ* conversion C_12_SM into C_12_Cer-1-P sequentially results in the formation of domains with higher lipid packing, the extrusion of tubular structures and, finally, in destabilization of the vesicular structure of the GUV (collapse), although a few remaining vesicles fully in a *s_o_*-like phase (high LAURDAN GP) are observed. This sequence of events would explain: i) the increase in polydispersity observed in DLS (Fig. 5) that progressively increases the noise in the LAURDAN GP measurements (Fig. 9) and, ii) the faster sedimentation of material from initially monodisperse LUV suspensions (Fig. 8). Membrane fusion was not studied in GUVs because the yield is relatively low and the density of observable vesicles is enough to allow visualization of events at the level of single vesicles.

The conversion of C_12_SM in LUVs by high enzyme concentration (≥1 µg/ml) is almost quantitative within a few minutes as evidenced by thin layer chromatography (data not shown), indicating that nearly all substrate molecules are eventually available for enzymatic hydrolysis. At lower enzyme concentrations (50–100 ng/ml) nearly complete hydrolysis could not be observed for up to two hours. In all experiments with LUVs, the concentration of C_12_SM was kept constant at 0.25 mM. Assuming 100 nm vesicles and a molecular area per lipid of 50 Å^2^, this corresponds to ∼126,000 C_12_SM molecules per vesicle. At a concentration of 1 µg/ml of SMD (∼32 kDa), there are on average 16 SMD molecules/LUV. Therefore, it is reasonable to suppose that at any given moment the number of SMD molecules acting on a single vesicle will follow some probability distribution and, therefore, that the morphological transformations may be heterogeneous. Coupled to the component of “scooting" apparent in our experiments, the course followed by any given vesicle would be determined largely by the number of enzyme molecules initially attached to and acting on it. It is also conceivable that the location of enzymes on the surface of a given vesicle may determine the sequence, and therefore the nature, of its morphological fate. An interesting feature of the morphological changes observed is the extrusion of tubular structures by some vesicles. These structures have a higher LAURDAN GP than the untreated vesicles, suggesting that they incorporate C_12_Cer-1-P. It is unclear, however, whether they are *bona fide* lamellar structures. Considering the destabilization of unilamellar vesicles by high molar fractions of C_12_Cer-1-P, premixed or enzyme generated, it is reasonable to hypothesize that this lipid favors non-lamellar structures, e.g. hexagonal phases or tubular micelles. This is consistent with the facilitation of the lamellar to hexagonal phase transition in Cer-1-P/ dielaidoylphosphatidyl-ethanolamine mixtures [27] and the increase in permeability in SM/cholesterol vesicles by SMD [61]. It is clear that this phenomenon adds to the structural complexity of the system beyond simple fusion events that would result in just larger vesicles.

### SMD activity on membranes containing Cer-1-P and cholesterol

Progressive increase in the fraction of C_12_Cer-1-P up to 23 mol% in LUVs used as substrate has two effects: it reduces the overall measured membrane dipolar relaxation (Fig. 2) and decreases maximal initial velocity of the enzyme. Importantly, in this range of C_12_Cer-1-P molar fractions, stable unilamellar vesicles exist in the *initial conditions* and, just like pure C_12_SM vesicles, are destabilized by the enzyme (data not shown). The reduced dipolar relaxation of the pre-mixed samples means progressive dehydration of the lamellar arrangement and is a consequence of the coexistence of *s_o_* domains with *l_d_* domains of mixed composition [38]. This interpretation is consistent with a progressive reduction of enzyme activity since a hydrolytic event requires at least one water molecule.

The 50 mol% substrate-product mixture, however, deserves separate consideration. Although the increase in the bulk LAURDAN GP could be interpreted as additional incorporation of the product into a lamellar phase, the DLS data (Fig. 12B) and the LAURDAN GP value of the sedimented material (Fig. 8D) show that this is not the case. There seems to be a point, somewhere above 23 mol% C_12_Cer-1-P, beyond which no more of this lipid can be incorporated into a bilayer and a multiplicity of different structures is generated. This idea is consistent with the incapacity to generate stable unilamellar vesicles from this mixture and pure C_12_Cer-1-P. The recovery of enzyme activity is therefore likely to be due to the action of SMD on a C_12_SM-rich sub-population (such as those in Fig. 8D) available once the unilamellar vesicle population is destabilized.

Cholesterol impacts the behavior of the system in several ways and the response of the enzyme is also non-linear (Fig. 13A). For this reason, we will address first the 9 and 23 mol% mixtures. In the first instance, the initial size distribution of the vesicles is maintained (DLS experiments in Fig. 13). The effect of 9 mol% cholesterol on reaction rate is negligible but the rate reaches a minimum at 23 mol%. In both cases the stability of the vesicles is not affected by SMD. In this concentration range, LAURDAN GP measurements before enzyme addition indicate that dipolar relaxation decreases, consistent with the progressive transition to a *l_o_* phase observed in glycerophospholipids [62]. These observations also support the notion (see above) that the initial state of hydration of the substrate modulates enzymatic activity. From the point of view of vesicle stability, cholesterol seems to offset the gross destabilizing effect of the product generated *in situ* by the enzyme.

The non-linearity of the effect on reaction rate in the 50 mol% C_12_SM∶cholesterol mixture, however, is not simple to explain. For this mixture without enzyme, the LAURDAN GP increases as expected but the reaction rate is higher than that of the 23 mol%. Also, the stability of the vesicles is different from that of the 50 mol% substrate∶product mixture. At present, we do not understand the underlying mechanism, but it is clear that there is an increase in the availability of the substrate to SMD. Although there is a dearth of detailed physicochemical characterizations of shorter chain sphingomyelins (such as C_12_SM) with cholesterol, we see two possibilities:

- In bilayers of egg SM mixed with up to 50 mol% cholesterol, vesicles incorporate only 43 mol% cholesterol [63]. However, beyond this threshold the formation of cholesterol crystallites is possible [64]. If this were true in the C_12_SM system, it raises again the point made above that the system may not be homogeneous anymore, i.e. generating subpopulations of structures in which the substrate is more readily available.
- Parasassi et al [50] reported on particular cholesterol concentrations at which abrupt variation of glycerophospholipid-containing membrane properties can be observed using the GP function. They proposed that the formation of ordered molecular microdomains at critical cholesterol concentrations, can explain the occurrence of these discontinuities in the GP function [65]. These discontinuities could reflect local heterogeneities with higher substrate availability. However, it is not known whether this phenomenon also occurs in C_12_SM membranes.

Finally, the structural consequences of SMD action on GUVs composed of DOPC∶egg SM∶cholesterol is relevant to the discussion since this mixture increases the level of initial compositional complexity of our membrane models. The main effect observed is that the coexistence of micron-sized *l_o_/l_d_* domains is strongly affected by enzymatic generation of Cer-1-P. In contrast to C_12_SM GUVs, there is no shrinking, no domain formation and no extrusion of tubes also supporting the idea that cholesterol offsets the structural effects of the product. Two interpretations of the disappearance of domains are possible: i) the domains vanish, indicating that the favored interaction between SM and cholesterol that dictates the formation of *l_o_* domains in the ternary mixture is strongly diminished by Cer-1-P; alternatively ii) the size of these domains is reduced (from micro- to nanoscopic) by an effect of the product on the domain line tension, falling below the ∼300 nm resolution of our microscope. Although the influence of higher compositional complexity on the effects of Cer-1-P awaits systematic study, our results show a very clear fact: this lipid can generate microscopic domains in one context and destroy them in another, but in neither case does its appearance leave the supramolecular arrangements unaffected. If the enzymatic generation of Cer-1-P is compared to that of ceramide (the product of sphingomyelinase C on SM) in the DOPC∶egg SM∶cholesterol system, a simple change in the polar head group results in dramatically different supramolecular behaviors. In coexisting *l_o_*/*l_d_* systems, the *in situ* generation of ceramide does not destroy microscopic domains but rather forms crystalline domains within the *l_o_* regions [66].

Our observations show that *in situ* generation of Cer-1-P has profound effects on membrane structure, suggesting that its role may extend beyond that of a classical second messenger.

### Conclusions

1. The action of SMD is dependent on the initial packing of the model membranes containing the substrate sphingomyelin and *in situ* generation of Cer-1-P has profound effects on their supramolecular properties.
2. The effects of Cer-1-P are dependent on the composition of the membrane. We hypothesize that SMD may have different effects on different cellular targets.
3. The fact that SMD has a well established activity on lysophospholipids does not mean that its action on sphingomyelin can be discarded in understanding loxoscelism.
